# Isolated Pulmonary Valve Endocarditis Complicated With Septic Emboli to the Lung Causing Pneumothorax, Pneumonia, and Sepsis in an Intravenous Drug Abuser

**DOI:** 10.1177/2324709613514566

**Published:** 2013-11-28

**Authors:** Deephak Swaminath, Yasir Yaqub, Roshni Narayanan, Ralph F. Paone, Kenneth Nugent, Aliakbar Arvandi

**Affiliations:** 1 Texas Tech Health Sciences Center, Lubbock, TX, USA

**Keywords:** endocarditis, pulmonary valve endocarditis, right-sided endocarditis

## Abstract

Intravenous drug users are at increased risk for developing right-sided infective endocarditis involving the tricuspid and pulmonary valves. Isolated pulmonary valve endocarditis in intravenous drug users is very rare, and these patients often have more complications, such as pulmonary embolism, sepsis, and pneumonia. We report a case with pulmonary valve endocarditis and extensive pulmonary complications, including sepsis, septic emboli, pneumonia, and pneumothorax. Early identification of pulmonic valve endocarditis and treatment with appropriate antibiotics with or without surgical management should provide better outcomes, and clinicians need to think about pulmonary valve endocarditis in patients with complex respiratory presentations.

Isolated pulmonary valve infectious endocarditis occurs infrequently but can present with significant respiratory complications. We report a case of *Staphylococcal aureus* pulmonary valve endocarditis complicated by pleural effusion, pneumothorax, multiple cavitary infiltrates, and acute respiratory failure.

## Case Report

**Figure 1.**
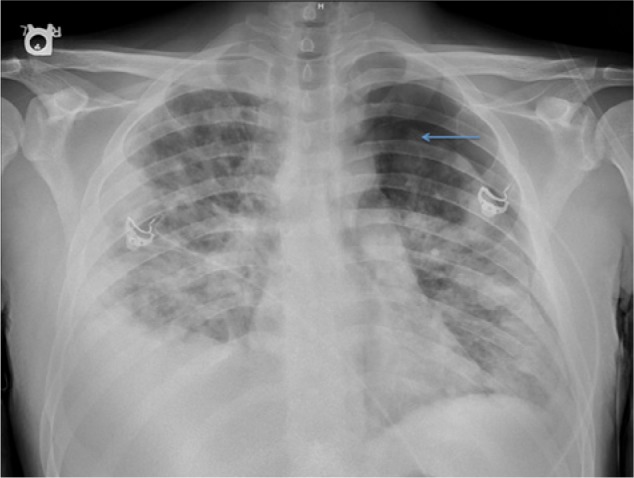
Chest x-ray showing bilateral infiltrates, left pneumothorax, and right pleural effusion. Pneumothorax (marked as a blue arrow) in the left lung.

A 25-year-old man presented to the emergency department with fever and shortness of breath. He had had increasing shortness of breath at rest for the past 2 days associated with multiple episodes of hemoptysis and fever. He also complained of diarrhea, nausea, vomiting, and fatigue for the past 5 days. His past medical history was significant for intravenous drug abuse and a recent soft tissue infection in the distal humeral region of his left arm, which was incised and drained but treated with an incomplete antibiotic course due noncompliance. On admission, he had severe respiratory distress with a respiratory rate of 32 breaths/min and required a non-rebreathing mask for oxygen supplementation. His initial chest x-ray revealed multifocal bilateral infiltrates and left-side pneumothorax (Figure 1). A chest tube was placed immediately on the left side.

**Figure 2.**
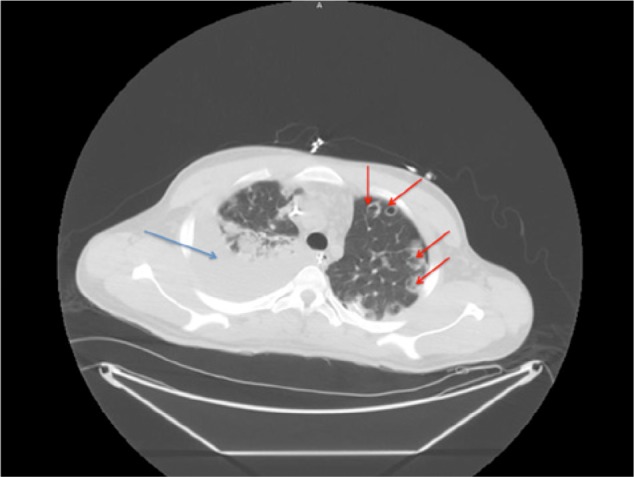
Computed tomography scan of the chest showing infiltrate (marked as blue arrow) and multiple small cavitatory lesions (marked as red arrow) in bilateral lung.

**Figure 3.**
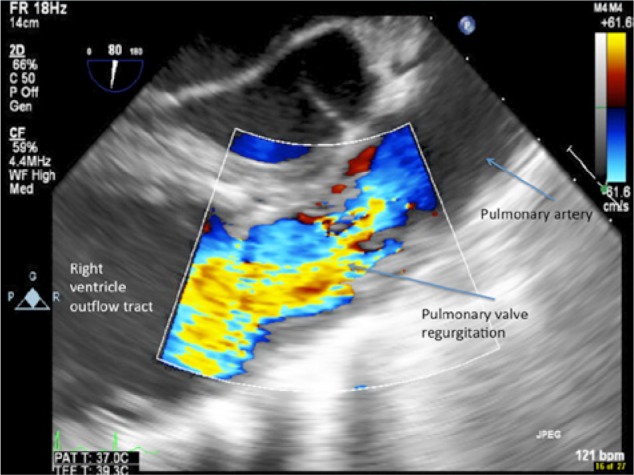
Transesophageal echocardiogram pulmonary outflow view showing severe pulmonary insufficiency.

**Figure 4.**
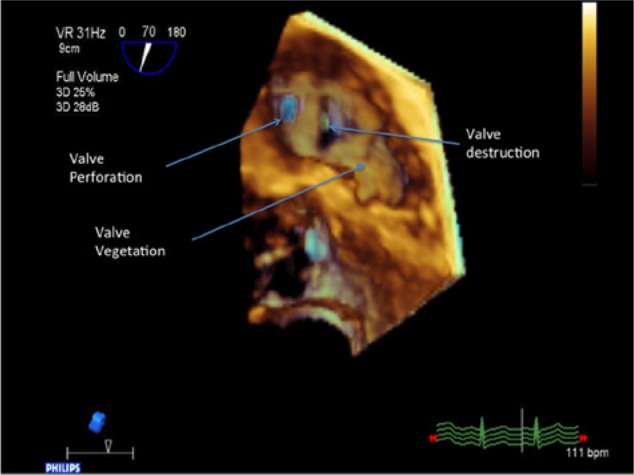
Transesophageal echocardiogram with 3D pulmonary valve with multiple valve perforation visible.

He was admitted to the medical intensive care unit and subsequently intubated due to respiratory failure. Initial lab work showed a white blood cell count of 46 400/µL and a platelet count of 64 000/µL. The patient became hemodynamically unstable and was started on vasopressors and empiric antibiotics. His chest x-ray and computed tomography scan (Figure 2) of the chest revealed patchy airspace consolidation and numerous cavitary infiltrates in both lungs. Blood cultures were positive for Gram-positive cocci in chains and clusters. A transthoracic echocardiogram showed severe pulmonic regurgitation with thickened leaflets and severe pulmonary hypertension (Figures 3 and 4). A transesophageal echocardiogram revealed vegetations on all the pulmonic valve leaflets, valve perforation, and severe regurgitation (Figure 4). The patient was started on rifampin and penicillin G.

Cardiothoracic surgery was consulted for pulmonic valve replacement, and the patient’s pulmonary valve and the vegetations were completely removed and replaced with a #25 pericardial valve. This pericardial valve was chosen because it lasts longer in the pulmonary position than either a homograft or a porcine valve. At the end of the procedure, there was no pulmonary valve regurgitation and no perivalvular leak. The overall left ventricular function was preserved. He developed severe coagulopathy during the postoperative period and required multiple units of fresh frozen plasma, platelets, and cryoprecipitate. He was successfully weaned off the ventilator after diuresis. The patient was treated with penicillin G and rifampin for 6 weeks based on culture and susceptibility results; repeat cultures were negative after starting penicillin G and rifampin. *Staphylococcus aureus* was grown from the valve removed during surgery, and susceptibility tests demonstrated that it was sensitive to penicillin G.

## Discussion

Infectious endocarditis of the pulmonic valve is seen in less than 1.5% to 2% of all cases of endocarditis and is usually associated with tricuspid valve endocarditis.^1^ Isolated pulmonic endocarditis is uncommon in adults, and only a few case series have been published about this disease.^2^ Most cases with pulmonic valve infectious endocarditis (PVIE) have occurred in children with congenital heart disease, such as a bicuspid valve.^3,4^ However, PVIE can also occur in patients with healthy hearts, especially in intravenous drug abusers. The literature includes 45 cases between 1960 and 2005.^5,6^ Review of this literature revealed that none of the cases had severe complications, such as pneumothorax and sepsis from septic emboli from the valve. Seventy-six percent of cases with right-sided infectious endocarditis (RSIE) are associated with drug abuse. In drug abusers, the tricuspid valve is usually involved (40% to 69%). Isolated involvement of the pulmonary valve is 10 times less frequent, and pulmonary valve involvement, when present, is usually associated with infection of the tricuspid valve.^7^ Other predisposing factors include central venous catheters, chronic alcoholism, extracardiac infections with bacteremia, and alterations in the host immune status.^2,5^ An autopsy study done on 9 PVIE patients revealed that the pulmonary valve was tricuspid in 6 cases, bicuspid in 2 cases, and unicuspid in 1 case. In the same study, concomitant thickening, shortening, perforations, or complete destruction of the cusps accompanied the pulmonic valve vegetations.^4^ Involvement of the right ventricular outflow tract or the main pulmonary artery was identified in 5 hearts. Five patients (55.6%) developed pulmonary complications related to the endocarditis.^4^

Most patients present with fever, shortness of breath, and pleuritic chest pain. A presentation consistent with pulmonary embolism with supporting radiographic and laboratory evidence has been observed. About 50% of these patients have a pulmonic regurgitant murmur on auscultation. Transthoracic echocardiography can detect pulmonic valve vegetations, but transesophageal echocardiogram is often needed to fully characterize the involvement. The bacterial pathogens are usually *Staphylococcus aureus*, coagulase-negative staphylococci, or Group B streptococci.^2,5,8-10^ Dialysis catheter–related endocarditis has been reported in a case of isolated pulmonic valve endocarditis caused by *Enterococcus faecalis*.^7^ *Pseudomonas aeruginosa* endocarditis was described in a case report and complete recovery of the patient was noted after surgical removal of the infected pulmonic valve.^11^ Few case reports of pulmonic valve endocarditis in pediatric population was described in the literature.^12,13^ A significant delay often occurs in the diagnosis of pulmonic valve endocarditis due to the lack of typical diagnostic symptoms and signs unlike cases of a mitral or aortic valve endocarditis.^14^ For example, the patient discussed in this case report presented with severe respiratory complications secondary to multiple septic emboli.

Antimicrobial therapy for suspected PVIE should be initiated immediately after adequate blood cultures have been obtained. Empirical therapy requires an antistaphylococcal agent with activity against methicillin-resistant *Staphylococcus aureus*, such as a combination of vancomycin and gentamicin or daptomycin alone. After culture and sensitivity results are available, antibiotics should be continued for 6 weeks. Surgery is indicated for RSIE in patients who have more than 3 weeks of persistent fever on a regimen of adequate antibiotic treatment, repetitive pulmonary emboli, vegetations larger than 20 mm, septic shock and documented RSIE, new onset renal and hepatic failure, complications after percutaneous removal of infected intracardiac wires, or secondary (right- or left-sided) valve endocarditis.^15,16^ Also, specific pathogens, including *Staphylococcus aureus*, Gram-negative bacilli, and fungi, need surgery early in the course of the disease.^15^ Stentless xenografts and pulmonary homografts are the most commonly used replacement procedures.^17^ There is also some evidence supporting the use of stented biologic valves or the bovine jugular vein (Contegra).^18^ A literature review on management of isolated pulmonary valve endocarditis involving previously normal hearts indicated that surgical therapy was required in 33% of the 36 identified patients. Surgical therapy included pulmonary valve replacement (33%) and pulmonary valvectomy (67%). The overall mortality was 19%, but there were no deaths in the surgical group, indicating that early surgical intervention might improve prognosis.^19^

In summary, isolated pulmonary valve infectious endocarditis is a rare condition in intravenous drug abusers. The key point from this case report is that diagnosis of the PSIE at an early stage of the disease should improve the outcome. Unlike the left-sided endocarditis, which presents with severe symptoms at the early stage of the disease, RSIE often presents with subtle symptoms in the early stage of disease. With the right diagnostic procedures, appropriate antibiotic treatment, and, if necessary, surgical intervention, both the short-term and long-term prognosis of the patient can be improved.
